# Qualitative exploration of public and smoker understanding of, and reactions to, an endgame solution to the tobacco epidemic

**DOI:** 10.1186/1471-2458-12-782

**Published:** 2012-09-13

**Authors:** Richard Edwards, Jo Peace, Marie Russell, Heather Gifford, George Thomson, Nick Wilson

**Affiliations:** 1 University of Otago, Wellington, Wellington, New Zealand; 2 Whakauae Research, Whanganui, New Zealand

## Abstract

**Background:**

There is increasing interest in ending the tobacco epidemic and in applying ‘endgame’ solutions to achieve that goal at national levels. We explored the understanding of, and reactions to, a tobacco-free vision and an endgame approach to tobacco control among New Zealand smokers and non-smokers.

**Methods:**

We recruited participants in four focus groups held in June 2009: Māori (indigenous people) smokers (n=7); non-Māori smokers (n=6); Māori non-smokers (n=7); and non-Māori non-smokers (n=4). Participants were from the city of Whanganui, New Zealand. We introduced to them the vision of a tobacco-free New Zealand and the concept of a semi-autonomous agency (Tobacco-Free Commission [TFC]) that would control the tobacco market as part of an endgame approach.

**Results:**

There was mostly strong support for the tobacco-free New Zealand vision among all groups of participants. The reason most commonly given for supporting the vision was to protect children from tobacco. Most participants stated that they understood the TFC concept and reacted positively to it. Nevertheless, rather than focusing on organisational or structural arrangements, participants tended to focus on supporting the specific measures which a future TFC might facilitate such as plain packaging of tobacco products. Various concerns were also raised around the TFC, particularly around the feasibility of its establishment.

**Conclusions:**

We were able to successfully communicate a complex and novel supply-side focused tobacco control policy intervention to smokers and non-smokers. The findings add to the evidence from national surveys that there is public support, including from smokers, for achieving a tobacco-free vision and using regulatory and policy measures to achieve it. Support for such measures may be enhanced if they are clearly communicated and explained with a rationale which stresses protecting children and future generations from tobacco smoking.

## Background

There is growing international interest in ‘endgame’ ideas and strategies in relation to tobacco products and tobacco control (see for example, themes in the March 2012 issue of the journal “Tobacco Control”) [1-4]. The tobacco endgame can refer to a goal such as achieving sales ban on tobacco products or a strategy – i.e., a coordinated approach and plan for achieving endgame goals. Tobacco control organisations and official bodies are increasingly advocating ambitious endgame goals. For example, in Finland a Tobacco Act was passed in 2010 which aimed to “put an end to the use of tobacco products in Finland” [5]. This Act followed a recommendation from The Cancer Society of Finland that Finland should be wholly smokefree by 2040 [6]. In New Zealand, the Tupeka Kore (tobacco-free) vision was launched by a range of concerned non-governmental organisations (NGOs) and advocacy groups in 2009. This proposed a target and a series of interventions to achieve close to zero tobacco smoking prevalence by 2020 [7]. Subsequently, the Māori Affairs Parliamentary Select Committee released a report recommending that New Zealand should be smokefree by 2025 [8], and the Government has since affirmed support for this goal [9].

In parallel with the emergence of endgame thinking, there has been increasing interest in supply-side approaches to tobacco control, such as retail-based interventions [10-13].This is an area of policy that has until recently received little attention, compared with demand reduction approaches such as mass media campaigns, tobacco tax increases and smoking cessation support. There have been few efforts to address the structure of the supply and distribution system for tobacco products. In most countries this is an unregulated market, in which tobacco companies manufacture and/or import and distribute tobacco products, and retailers sell tobacco products on a commercial basis with the aim of maximising sales and profits [14].

Some of the overarching mechanisms suggested for achieving endgame goals target the nature of the nicotine market and the structural arrangements for the regulation of nicotine delivery products. One suggested mechanism is to introduce regulatory authorities with sufficient powers to limit commercial activity, and achieve a healthier regulatory balance between smoked tobacco and pharmaceutical or other safer nicotine delivery products [15-18]. Others have argued that the tobacco product production and distribution systems should be changed, for example, through the creation of a not-for-profit distribution agency [19], making the tobacco industry a not-for-profit enterprise [14,20], or through a progressive reduction in the importation and release for sale of smoked tobacco products [21,22]. However, the increasing interest in such endgame approaches has seldom been reflected in the political agenda or national tobacco control strategies. The most dramatic exception is in Bhutan, where the sale of tobacco products was banned in 2004 [23].

The study we report here was carried out in New Zealand, where tobacco-related harm remains high. Following substantial declines in the 1970s and 1980s, recent trends have been for only a very gradual decline in smoking prevalence [24,25], which remains about 20% among adults, with much higher prevalences among Māori (indigenous New Zealanders) (above 40% prevalence), young adults and socio-economically disadvantaged groups [26]. Tobacco is not now grown commercially in New Zealand and is imported in raw form (for a single manufacturing plant for cigarettes) or is imported as manufactured cigarettes. Current regulation effectively eliminates advertising and sponsorship, requires indoor public areas to be smokefree, and mandates pictorial health warnings on packs. In addition, increased taxation (price) and social marketing have been used to try to reduce smoking and exposure to second-hand smoke. The cessation system includes a national telephone Quitline, considerable availability of subsidised nicotine replacement therapy products and some culturally specific cessation support programmes for Māori smokers.

Preliminary (unpublished) pilot work by the authors in 2008 suggested that radical endgame solutions are poorly understood by the public, media and policy makers, and may be difficult to communicate. It was therefore timely to investigate the feasibility, acceptability and methods of communicating these endgame approaches to tobacco control with these audiences.

In the first phase of this project we investigated views and understanding of public, health practitioners, media and policy makers to five innovative supply-side and endgame strategies to tobacco control. We found widespread support for the endgame goal and high levels of interest and engagement with the endgame approaches, with varying degrees of support for each proposed strategy [27].

We report here on phase two of this study in which we conducted focus groups to explore further with the public (smokers and non-smokers) views about (i) the vision of a tobacco-free New Zealand and (ii) one of the possible means to achieve it included in phase one of the project. This means was the creation of a not-for-profit distribution agency (or regulated market model) as proposed by Borland [19]. The aims of the focus group research were to (a) assess the extent and nature of support or opposition to the goal of a tobacco-free New Zealand; (b) assess the comprehensibility of the regulated market model; and (c) to explore views about its strengths and weaknesses.

## Methods

Following the first phase of the project, we identified one of the endgame approaches discussed as having particular promise for effectively addressing tobacco use in New Zealand. This was the introduction of a semi-autonomous agency to act as a monopoly purchaser of tobacco products, and control a not-for-profit supply and distribution system (the regulated market model). For the second phase of the project we named this agency the ‘Tobacco-Free Commission’ (TFC). With advice from a communications expert we developed information materials including a slide presentation outlining the operation and activities of the proposed Commission.

We recruited members of the public (n=24) to attend four focus groups in Whanganui in June 2009 comprising: Māori smokers (n=7); non-Māori smokers (n=6); Māori non-smokers (n=7); and non-Māori non-smokers (n=4). All participants were aged between 19 and 60 years and there were 11 males (7 smokers, 4 non-smokers) and 13 females (6 smokers and 7 non-smokers). Whanganui was selected as one of the team (HG) had particularly strong links with the community and this was desirable for good recruitment of Māori smokers. Potential participants were informed that they would be taking part in a discussion about “some innovative proposals for improving the way tobacco control policies are introduced and implemented, and how tobacco is distributed and marketed in New Zealand” and were asked to talk about “their views about how these proposals could be communicated most effectively to different audiences”.

**Figure 1:**
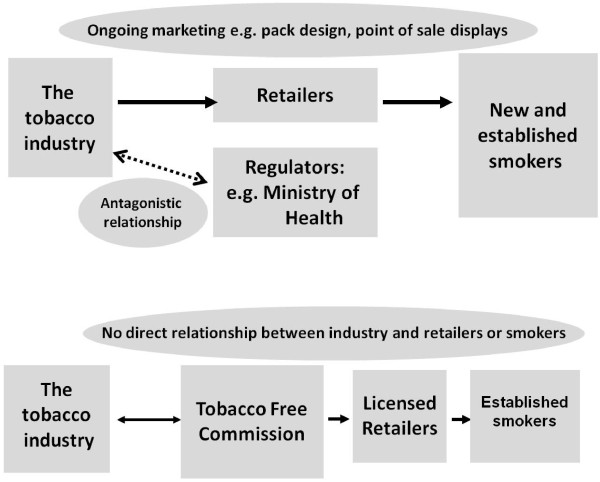
Summary slide used to describe the current tobacco supply system and the Tobacco Free Commission (adapted from slide used during presentation).

Although a written hand-out introducing the concept of the TFC had been prepared, on advice from Whakauae Research Services which recruited the participants, we did not distribute this prior to the discussion by the focus groups, and relied on a presentation by the first author (RE) to introduce and explain the operations of the proposed new agency. The presentation began with a description of the extent of the public health problem posed by tobacco in New Zealand and outlined the vision of a tobacco-free country in which children would be free from exposure to tobacco and smoking prevalence is close to zero. It was argued that new approaches would be needed to achieve the tobacco-free vision, and that one such approach was a TFC. This was described as a semi-autonomous and not-for-profit government agency with a public health mandate which would act as a monopoly purchaser and distributor of tobacco products (see Table 1 and Figure 1). The TFC would control the supply of tobacco, and facilitate and promote measures to reduce smoking prevalence. Examples of measures given included banning point-of-sale tobacco displays, introducing plain packaging, licensing and controlling the number of retailers that could sell tobacco and their proximity to schools, and mandating that tobacco retailers provide cessation support and sell cessation support products.

**Table 1:** Key components of the tobacco free commission as presented to focus group participants

**Tobacco Free Commission facets presented**	**Detail provided**
Purpose	To facilitate the achievement of the tobacco-free vision
Mode of operation	TFC mandate is to support and facilitate measures to reduce smoking prevalence not maximising sales or profit
	Independent of government
	Transparent governance structure
	Non-profit making
	Focus on controlling and reducing supply of tobacco products
	TFC to be disbanded once tobacco targets are reached.
Structure/relationships	Contractual relationship between TFC and tobacco industry (TFC commissions tobacco industry to provide supply of tobacco products)
	TFC supplies tobacco products to licensed tobacco retailers (no direct supply of retailers by tobacco industry)
Other possible measures which TFC could introduce/facilitate	Licensing tobacco retailers
	Controls over number and location of tobacco retailers
	Removal of point-of-sale tobacco product displays
	Tobacco retailers required to provide smoking cessation support and aids
	Plain packaging of tobacco products

The focus group interviews were conducted by two team members (RE and MR) and lasted between 60 and 94 min. All were audio-recorded with group members’ consent. The main areas of discussion were: views about the tobacco-free vision, understanding of the TFC idea, reactions to the TFC, and views about how the model was presented and communicated.

The focus group tapes were transcribed, and then the transcripts were reviewed and data coded into a matrix (by MR). Themes and sub-themes were identified inductively through thematic analysis. This involved the reading and re-reading of the transcripts and the coded sections from the focus group discussions. Following the initial coding and identification of themes, the transcripts and coded data were further scrutinised independently by two other team members (RE and JP) and a final set of themes agreed following further discussion. Quotes were identified to illustrate participants’ views, where these were judged to be particularly apt and succinct.

The study was reviewed and approved by the Department of Public Health, University of Otago, Wellington under the ‘Category B’ Ethics review process of the University of Otago. All participants gave written consent after reading an information sheet about the project. Participants were provided with $30 vouchers for a general store in recompense of their time.

## Results

All the focus groups worked well with a rich discussion and with good involvement of all participants. Results are presented for two main facets of the discussion – understanding of the TFC, and support for the Tobacco-Free Vision and establishing a TFC in order to achieve it.

### Participants’ understanding of a tobacco-free commission

Most participants stated that they understood the TFC concept as presented.

“[It’s] a great idea, it’s clear and well presented.” (Māori non-smoker)

With further probing a few participants explained the idea back to the facilitator. For example, one participant compared it to the regulated nature of pharmaceuticals and hence restricted availability of these. Another more limited description of the model was:

“So people can still get tobacco, but it’s in a less in your face kind of way, it’s regulated to a degree.” (non-Māori non-smoker)

Participants usually stated that they understood the idea, but it was not possible in the time available to investigate if each participant could explain it back to the facilitator, so the depth of understanding could not be fully gauged. A hint that maybe the TFC was not fully understood by some came from one participant, who responded to a question to the group about whether they understood the TFC:

“It would be hard to explain it to other people.” (Māori smoker)

### Support for the tobacco-free vision and tobacco-free commission

There was mostly strong support for the tobacco-free New Zealand vision among all groups of participants. The reason most commonly given for supporting the vision was to protect children.

“But for kids not to smoke I think is - it’s a great thing to bring in. I’d hate my kids to start smoking.” (non-Māori smoker)

Others supported the vision due to the need to reduce deaths caused by tobacco.

“I think it needs to be done. I think it’s necessary. When you look at the statistics of tobacco related deaths, it is-yeah, it is necessary.” (Māori smoker)

Two of the Māori smokers felt particularly strongly in support of the tobacco-free vision.

“Yeah. Just don’t fail. Just don’t. You know, make it work, make it happen.”

"“I also think too, we … have a responsibility to pass the message on to our families.”

Responses to the TFC idea were also generally positive although a lot of the support was expressed not as support for the TFC model and structure, but rather as support for specific measures which were mentioned in the presentation as among those which the TFC might facilitate, for example, plain (unbranded) packaging, licensing of and reducing the number of tobacco retailers, and removing point-of-sale retail displays:

“Definitely hiding them. Yeah, and making them de-labelled, is a good idea as well.”(Māori smoker)

“So I think the idea of getting smokes out of sight, and maybe restricting the amount of dairies, or - you know, things like that, dairies, plus close to schools, that would be good.” (non-Māori smoker)

“I like the idea of the plain packets of the cigarettes, and definitely handing out more information on its effects when people do buy it and that.” (non-Māori non-smoker)

“I think the packaging will make a – like it says, you’re drawn to certain designs and brands. And if it was all generic, I think given that, and putting it below eye level, there’s no real incentive, unless you really want to smoke”. (non-Māori non-smoker)

However, some did express strong and specific support for the TFC concept.

“Yeah, I support the idea. I wish this had come out years ago…” (Māori non-smoker)

Quitting smoking had changed the views of one participant.

“Well a year ago I wouldn’t have listened to this conversation, but now I do. I’ve given up smoking for six weeks. And I am glad I’m succeeding. Yeah, I agree with everything you said, I like that idea.” (Māori non-smoker)

Support also came from smokers who seemed committed to or resigned to continuing to smoke.

“I like the idea, but I know I’m cutting my own throat by agreeing with it. Because it’s working towards going against what I really really enjoy. But – so yeah, I think it would work. It’s a good idea.” (Māori smoker)

“I think it’s good, definitely a good idea…., you’re definitely not going to be able to stop smokers now, like people, … like us, but at least if you can do it, and make it happen, then you’ll stop other people from starting, you know. Like cos there’s just no hope for me.” (Māori smoker)

Again the reason for supporting the TFC approach was often brought back to the need to protect children from tobacco.

“I’ve got two young kids, I’d love them not to smoke…” (non-Māori smoker)

“I’d hate for my children to start smoking, or have to suffer like that – you know they get lung cancer …it’d (the TFC) be great.” (Māori non-smoker)

Another participant liked that the TFC would bring in stronger regulation whilst maintaining the choice of smokers to smoke.

“Well from a smoker’s perspective, they still get to smoke, if they choose to. From the Commission’s perspective, they get to re-educate and to regulate the supply of cigarettes, with the long term outcome being the reduction of smoking…. it’s sort of- everyone wins.” (non-Māori non-smoker)

Another liked the transparency and relative autonomy of the proposed TFC from government, retailers and from the industry.

“Well it’s a separate entity. It’s got no government influence, it’s got no influence from retailers … and from the tobacco industry … So therefore it is completely separate… it’s a- what do you call it, see-through, transparent.” (non-Māori smoker)

### Participant’s concerns and caveats

In phase one of the “Daring to Dream Project”, the most commonly identified barriers for introducing a TFC identified by senior policy makers, journalists, and public health practitioners were the perceived political and ethical difficulties of a government agency selling tobacco or nicotine [26]. The participants in this phase two study however, did not articulate this issue at all.

The most common concerns expressed related to the feasibility of establishing the TFC. Some participants noted that it would be vigorously opposed by the tobacco industry:

“…there’s tobacco companies. They’ve got so much power, so much money, they’re going to fight it tooth [and nail]…every step of the way.” (Māori non-smoker)

One participant argued that the tobacco industry would have to be neutralised in order to implement the TFC. Another questioned how the importation of tobacco could be controlled in practice. Several participants questioned the length of time needed to set up the TFC:

“Yeah, I mean how long is this going to take to actually get from here, to law, I mean that’s ten, twenty years, I don’t know.” (Māori non-smoker)

One participant questioned whether it could be achieved rapidly, and if not, whether it would be better just to implement some of the ideas discussed in the presentation like plain packaging, rather than putting energy into establishing the TFC.

Some participants argued that the key issue was whether the idea could be ‘sold’ to the government, one noted that selling the idea of an agency buying and selling tobacco was very radical.

“So you’re trying to market this to government … cos without government it’s not going to happen, is it? They’re the ones who’ll have to put some laws in place for it to happen, and you’ve got to sell that to the parties, different parties in parliament. The biggest message… the hardest one … will be the actual purchasing of tobacco. I can see … the plain packet stuff, that’ll be easy to sell that to the government. But the concept of trying to cut smoking by buying smoking [the smoking market] is a really out-there concept.” (Māori smoker)

“… if you can get the government to come forward, and help promote it, and support this, I think it can work – I believe it can work.” (Māori non-smoker)

Another set of concerns were expressed in relation to the structure and composition of the TFC. Several participants discussed the importance of getting the governance and membership right. For example, one Māori smoker felt that decisions about who was appointed as commissioners could become a ‘*political game’* and that governance had to be sorted out before the operational arrangements. Other participants argued that it was important that members of the tobacco industry and politicians weren’t included and others argued that smokers should be:

“Yeah, you got to have a good group of people, from different – all areas, I mean you got to have smokers on it as well. Cos like non-smokers are sitting there going …smoking’s bad, blah blah blah, but … smokers… they’re the ones that are buy[ing] them, so you’re better to get their input as well.” (non-Māori smoker)

One participant noted that it was important that bureaucracy be kept to a minimum:

“Just, you know, keep your bureaucracy down to a minimum. Don’t make it another body that swallows our taxes.” (Māori smoker)

Another concern expressed in the non-Māori smoker group was that the TFC might result in an increase in the black market or home-growing of tobacco. One smoker argued that he would simply grow his own tobacco if it was no longer available for sale.

“It won’t worry me, cos at the end of the day, if they stopped it, and banned it, I’d just start growing my [expletive] own.” (non-Māori smoker)

Finally, some concerns were expressed by smokers, especially the Māori smokers, in response to the specific measures mentioned in the presentation that the TFC might introduce. The central theme was how specific measures might disadvantage them as an individual smoker. For example, several of the Māori smoker focus group participants held strong preferences for the brand they smoked and felt that brands tasted differently. Some were concerned that plain packaging would mean that they would not be able to identify their preferred brand, or it would no longer be available.

“People … don’t just go into the shop and buy like oh, any old crap, you know. You go in there and you know what you want.” (Māori smoker)

“So you know, if you’re going to de-label them, you’ve got to make sure that it’s still the same tobacco.” (Māori smoker)

Some smokers worried about the price of their cigarettes increasing, and argued that due to the simpler packaging, prices should fall instead.

“Oh, it’s a good idea. As long as it’s, you know, it’s not the price going up. That’s part of the price, the packaging. I mean that’s why you have no frills, and no brand.” (non-Māori smoker)

## Discussion and conclusion

We were able to successfully communicate a complex and novel supply-side focused tobacco control policy intervention to a diverse group of smokers and non-smokers. The articulated support from focus group members adds to the evidence from national surveys that there is public support for achieving a tobacco-free vision and for a wide range of additional tobacco control interventions. Our findings also tentatively suggest that support for such measures may be enhanced if they are clearly communicated with the policy intervention fully explained and a clearly communicated rationale which stresses protecting children and future generations from tobacco smoking.

Strengths of this qualitative study include the diverse range of opinions sought and the in-depth exploration of ideas that was possible within the focus groups. The inclusion of Māori focus groups ensured that the voice of people from communities with the highest smoking prevalence was included in the research. In addition, the concepts discussed were new to all of the participants, and so their thinking was relatively uninfluenced by media or political discourse on the issues.

While focus group methods can have limitations (such as dominance by a few individuals or “group think” processes) these groups appeared to function well with no obvious problems of this sort. Focus groups allowed us to assess understanding and support for the tobacco-free vision and TFC in a group setting, where participants were able to benefit from the interaction with others. Further research using in-depth interviews would provide additional and complementary data for triangulation of these findings. A possible limitation was that all the focus groups were facilitated by two Pakeha (European origin) researchers, which may have influenced the responses for the Māori focus groups. However, we note that (i) focus group participants were very forthcoming in their views; (ii) a local Māori research group organised recruitment to the focus groups and (iii) a team member (HG) was a high profile member of the local Māori community.

Generalisability is not a relevant issue for a qualitative study that is focused on in-depth understanding of the topic of interest. Even so, a possible limitation is that these focus group participants were from a single geographical area and there may be iwi-specific variation in views on tobacco control for Māori participants (given heterogeneity in such issues as the tobacco or smoke-free status of marae and local Māori leadership). However, additional support for these findings comes from evidence from national surveys in New Zealand that there is public support, including from smokers, for achieving a tobacco-free vision and using regulatory and policy measures to achieve it. This survey evidence includes high levels of support for an end to tobacco sales in 10 years time, among New Zealand smokers and general population [28-31].

The concerns and caveats raised by participants were largely about details of implementation (e.g., concerns about selling the idea to government, over-coming tobacco company opposition, determining the control group for the TFC, and controlling the black market) rather than opposition to the tobacco-free vision or the TFC idea in itself. Some of the issues raised are unlikely to be a major concern – for example, the composition of the TFC control group could presumably be worked through (as with other successful stand-alone government agencies in New Zealand e.g., the agency which purchases pharmaceuticals for the public health sector, “Pharmac”). Similarly, the black market is unlikely to be more than a minor problem in New Zealand due to its relative geographic isolation, very strong border controls (mainly for biosecurity reasons), and the difficulties with growing and curing tobacco in the country. However, additional work will undoubtedly be needed to develop detailed policy options and implementation plans for whatever endgame measures are adopted by government.

Although some participants discussed the strengths and perceived weaknesses of the TFC model, others focused more on discussing specific measures that might be introduced by the TFC, particularly plain packaging and removing point-of-sale displays. It may be that the TFC idea was somewhat too abstract or complex for some participants and more difficult to engage with than discrete tobacco control interventions. This suggests that communicating the TFC idea to the public will require a very clear, simple and engaging approach.

Further qualitative and quantitative research on the topic will be important. This could include exploring support for the tobacco-free vision, and researching policies and strategies to achieve support among smokers and non-smokers. To ensure that an equity lens is applied to research, policy and practice, future work should include a strong focus on research by and with members of communities most affected by smoking, for New Zealand: Māori, Pacific peoples, and those in deprived communities. Such research should help ensure that the level of public support is understood and monitored over time. It could provide advocates with evidence to inform political leaders of the political feasibility and importance of action to achieve the tobacco endgame. Nevertheless, in some jurisdictions it is intervention research which may be more appropriate – ie, a small island nation could introduce a ban on tobacco imports and then focus its research effort on studying the impact and the effectiveness of countermeasures to smuggling. A variety of other ‘endgame’ strategies have been proposed such as the ‘sinking lid’ [22] and creating a tobacco-free generation by increasing the legal age of purchase each year [32]. These and other approaches could be the subject of future research to explore smoker and non-smoker support.

In conclusion, our findings add to the evidence that endgame visions of a tobacco-free future have resonance with this public, including with smokers. These findings should suggest to advocates, practitioners, researchers and policy makers that these ideas should benefit from further research to explore their acceptability, methods of communication and framing, as well as further detailed policy analysis work on endgame options and thorough evaluation as endgame approaches are implemented.

## Competing interests

The authors declare that they have no competing interests, though for completeness note that RE, GT, HG and NW have carried out occasional consultancy work for health related NGOs and government agencies.

## Authors’ contributions

RE was the principal investigator for the Daring to Dream research project, assisted with the analysis and wrote the first and subsequent draft of this manuscript. MR carried out the bulk of the day to day work on the project, including organising and carrying out data collection and leading the initial data analysis. JP assisted with the analysis and editing of drafts of the paper. RE, NW, GT and HG contributed to the initial conceptualisation of the research, and to the design and implementation of the study at every stage. All authors took part in discussions about the scope and content of this paper, and contributed comments and text to each draft. All contributing authors have read and approved the final manuscript.

## Pre-publication history

The pre-publication history for this paper can be accessed here:

http://www.biomedcentral.com/1471-2458/12/782/prepub
